# Genome-Wide Association Study to Identify Candidate Loci for Biomass Formation Under Water Deficit in Perennial Ryegrass

**DOI:** 10.3389/fpls.2020.570204

**Published:** 2020-12-08

**Authors:** Kristina Jaškūnė, Andrius Aleliūnas, Gražina Statkevičiūtė, Vilma Kemešytė, Bruno Studer, Steven Yates

**Affiliations:** ^1^Laboratory of Genetics and Physiology, Institute of Agriculture, Lithuanian Research Centre for Agriculture and Forestry, Akademija, Lithuania; ^2^Department of Grass Breeding, Institute of Agriculture, Lithuanian Research Centre for Agriculture and Forestry, Akademija, Lithuania; ^3^Department of Environmental Systems Science, Molecular Plant Breeding, Institute of Agricultural Sciences, ETH Zurich, Zurich, Switzerland

**Keywords:** leaf growth, drought tolerance, genome-wide association study, dynamic phenotyping, *Lolium perenne* L., Phytochrome B, MYB41

## Abstract

Global warming is predicted to impact many agricultural areas, which will suffer from reduced water availability. Due to precipitation changes, mild summer droughts are expected to become more frequent, even in temperate regions. For perennial ryegrass (*Lolium perenne* L.), an important forage grass of the *Poaceae* family, leaf growth is a crucial factor determining biomass accumulation and hence forage yield. Although leaf elongation has been shown to be temperature-dependent under normal conditions, the genetic regulation of leaf growth under water deficit in perennial ryegrass is poorly understood. Herein, we evaluated the response to water deprivation in a diverse panel of perennial ryegrass genotypes, employing a high-precision phenotyping platform. The study revealed phenotypic variation for growth-related traits and significant (*P* < 0.05) differences in leaf growth under normal conditions within the subgroups of turf and forage type cultivars. The phenotypic data was combined with genotypic variants identified using genotyping-by-sequencing to conduct a genome-wide association study (GWAS). Using GWAS, we identified DNA polymorphisms significantly associated with leaf growth reduction under water deprivation. These polymorphisms were adjacent to genes predicted to encode for phytochrome B and a MYB41 transcription factor. The result obtained in the present study will increase our understanding on the complex molecular mechanisms involved in plant growth under water deficit. Moreover, the single nucleotide polymorphism (SNP) markers identified will serve as a valuable resource in future breeding programs to select for enhanced biomass formation under mild summer drought conditions.

## Introduction

Abiotic stress has detrimental effects on agriculture worldwide, threatening food security as huge yield losses are already being reported (Daryanto et al., 2016; Fahad et al., 2017). Moreover, concerns are rising about the effect climate shifts may impose on grassland productivity, and consequently feed production (Ergon et al., 2018). Climate models predict an increase in dry spells in the future, the key components being water deficit caused by the changes in precipitation patterns and temperature increase (Boyer, 1982; Lobell and Field, 2007; Salekdeh et al., 2009; Moore and Lobell, 2015).

Drought is a major limitation to crop productivity, crop irrigation consumes large amounts of water resources (Condon et al., 2004). Plant breeding for drought tolerant cultivars is considered as an important measures to mitigate the effects of drought on food and feed production, however, direct selection for yield under water stress resulted in limited genetic gain for drought tolerance (Hu and Xiong, 2014; Kumar et al., 2014; Mwadzingeni et al., 2016; Sandhu and Kumar, 2017). Plant phenotypes are inherently complex, reflecting the interactions of the genotype with environmental factors (Forsman, 2014). These interactions influence plant growth and development, which affect biomass accumulation and hence yield formation. A very important aspect to consider when breeding for drought tolerant crops is the severity and duration of the stress, as increased survival under severe drought does not mean improved performance under mild drought (Skirycz et al., 2011). When faced with water limitation during summer droughts in temperate environments, plants might employ alternative strategies, like drought escape, and drought avoidance (Kooyers, 2015). In either case, the outcome is reduced vegetative growth and biomass accumulation (Farooq et al., 2012). Plants can adapt to water stress in different ways, they might choose between survival, meaning reduced water use and growth halt, or continued slow growth (Claeys and Inzé, 2013). The latter option offers a competitive advantage in biomass accumulation, assuming the stress is temporary but can threaten survival if the stress persists. From a practical point of view, aiming to obtain stable yield under mild drought, a forage grass cultivar continuing slower growth after sensing the water deprivation is preferable to the one that opts for the survival strategy and halts its growth early.

Throughout the decades of perennial ryegrass (*Lolium perenne* L.) breeding, progress has been made in many quality-related traits (Humphreys et al., 2010; Sampoux et al., 2011, 2013). Drought adaptation of cultivars and natural ecotypes, reflected both in aboveground biomass production under field conditions and controlled environments, has been the focus of many studies (Korte and Chu, 1983; Kemesyte et al., 2017; Bothe et al., 2018; Lee et al., 2019). However, most of the earlier studies used destructive phenotyping methods or were limited to measuring the traits at start and end of the stress. This might lead to missing important quantitative trait loci (QTL) in association studies (Bac-Molenaar et al., 2016) as well as limited understanding of the overall complexity of temporal plant response to the environment. Recently, Yates et al. (2019) described a dynamic phenotyping method as a non-destructive and precise diagnostic tool to profile leaf growth under water deficit stress *in situ.* This method allows modeling leaf growth and pinpointing when a plant reduces and arrests leaf growth due to water deficit and thus has the potential to unravel the genetic mechanisms underlying these responses by QTL analysis. In this study, we aimed at: (i) determining the genotypic response of leaf growth to temperature and soil water availability, (ii) identifying genome-wide DNA sequence polymorphisms using genotyping-by-sequencing (GBS) for population description, and (iii) g genetic loci associated with the response to water deficit stress by performing genome-wide association study (GWAS).

## Materials and Methods

### Plant Material and Growth Conditions

A perennial ryegrass association panel consisting of 197 genotypes was used throughout the water deficit experiments. The panel was chosen on the basis of earlier experiments for drought (Jonavičienė et al., 2014) and freezing tolerance (Aleliūnas et al., 2015), where results revealed a high genotypic diversity among the panel individuals as well as a high phenotypic variability for drought-related traits. Most of the cultivars were of European origin, except one genotype coming from Japan, one genotype from New Zealand and six genotypes from the United States. The majority of ecotypes were of Lithuanian and Ukrainian origin, 35 and 55, respectively, but a few of the ecotypes were from Latvia (two), Poland (two), Slovakia (two), Denmark (one), and the Kaliningrad Region of Russian Federation (ten). A detailed description of the association mapping panel is presented in Supplementary Tables 1, 2.

For the leaf growth under water deficit experiment, plants were vegetatively propagated into four clonal replicates, each consisting of 20 tillers, and grown in plastic pots (ø15 cm and 12 cm height) filled with 450 *g* of commercial potting mix substrate (“Spezialmischung 209,” RICOTER Erdaufbereitung AG, Aarberg, Switzerland) under regular irrigation and fertilization in a greenhouse. 4 to 6 weeks after clonal propagation, plants were transferred into a climate chamber (Conviron, 86 Winnipeg, Canada) under controlled conditions with a light/dark photoperiod of 16/8 h and a light intensity of 275 μmol photosynthetically active radiation (PAR) m^–2^ s^–1^. The climate chamber was equipped with a 2:1 mixture of fluorescent lamps of two types (T5 FQ 54W/840 HO, Osram GmbH, Munich, Germany and T5 FH054W/GRO G5 F 54W, Havells Sylvania Europe Ltd, London, United Kingdom). The day/night temperature was 25/15°C, and relative air humidity was set to 50%.

### Water Deficit Treatment and Leaf Growth Measurements

To measure the phenotypic response to water deficit, leaf growth was assessed non-destructively using a largely automated phenotyping platform as described by Yates et al. (2019). Briefly, the tip of a young growing leaf was attached with a hair pin to a string and kept taut using weights of 20 *g*. White plastic beads (ø20 mm, 7 *g*) were threaded onto the strings and placed on the growth array to provide artificial landmarks for image-based marker tracking. Images of the growth array were taken every 2 min with a LupusNET HD camera (LUPUS-Electronics^®^ Gmbh, Landau, Germany) and analyzed with the LLT software (Nagelmüller et al., 2016).

To induce a water deficit stress, perennial ryegrass plants were deprived of water for 130 h. After the stress treatment, plants were re-watered and grown for additional 35 h to confirm that water limitation was the cause of leaf growth arrest, but not leaf age. Soil moisture deficit was measured using an integrated wireless microclimate sensing system (WiFi Plant sensor, Koubachi, Switzerland). Each sensor was calibrated individually and soil moisture data was measured at a depth of 7 cm every 4 h. The meristem temperatures of six plants per experiment were measured with a *K* type thermocouple (GTF 300, Greisinger, Germany; ø 0.2 mm) inserted into the tiller at meristem height.

The statistical analysis was implemented with the open source R statistical environment (version 3.1.0; R Development Core Team, 2005). Leaf growth under water limitation was estimated using the TriPhase function described by Yates et al. (2019). The function dissects complex growth processes into qualitative traits, described as leaf thermal growth (a), leaf growth reduction point (Σ), and the leaf growth arrest point (σ). The average phenotype for each trait per genotype was determined using least-square means implemented in “lsmeans” package for R (Lenth, 2016). Student’s *t*-test was used to compare the means of different growth traits and the Pearson’s correlation coefficients between different phenotypes were estimated. Broad sense heritability of the traits was determined using the “heritability” package of R (Kruijer et al., 2014).

### Genotyping-by-Sequencing and SNP Discovery

High-molecular-weight DNA was extracted from fresh leaf material using the method described by Mayjonade et al. (2016). The DNA was quality checked on a 1% agarose gel and quantified with a NanoDrop 2000 spectrophotometer (Thermo Fisher Scientific, Waltham, MA, United States). The association panel was divided into three groups with equal number of individuals in each to produce GBS libraries. One hundred ng of DNA of each individual was digested by *Pst*I (Thermo Fisher Scientific, Waltham, MA, United States) and then unique barcodes were ligated using T4 DNA ligase (Thermo Fisher Scientific, Waltham, MA, United States). The libraries were then pooled into a single tube and PCR-amplified using a common primer with a HiSeq adapter. After quality checking and cleaning using Qiagen spin columns (QIAGEN Sciences Inc, United States), the samples were sequenced by 126 bp single-end reads on a Illumina HiSeq 2500 (Illumina, Inc, San Diego, United States) sequencing system at the Functional Genomics Center Zurich (Zurich, Switzerland).

The quality of raw sequence data was checked using FastQC (v0.11, DePristo et al., 2011). After the initial quality check, reads of 164 genotypes were demultiplexed into separate files using saber^1^ with no mismatches allowed. Illumina adapter sequences were removed using Cutadapt (v1.18; Martin, 2011) and reads shorter than 40 bp were discarded. The reads were mapped to the perennial ryegrass draft genome assembly (Byrne et al., 2015) using BWA-MEM (Li, 2013). Bi-allelic variants were called using Genome Analysis Toolkits (GATK, v.3.8-1-0, McKenna et al., 2010) and then hard-filtered using vcftools (v.0.1.14, Auton et al., 2011) with minimum depth of 5 and minimum quality (GQ) score of 40. After removing single nucleotide polymorphisms (SNPs) with low-quality scores, variant sites were additionally filtered on allele frequencies by excluding variants with a minor allele frequency (MAF) below 0.05. Afterward, variant sites with more than 50% of missing data in the population were excluded.

### Linkage Disequilibrium, Population Structure and GWAS

Linkage disequilibrium (LD) in the association panel was expressed as squared Pearson’s correlation coefficient (*r*^2^) on all the marker-tagged scaffolds larger than 20 kbp. For LD analysis, SNPs with an estimated MAF lower than 0.05 were removed. Squared correlation coefficient between the variant sites was evaluated using vcftools (v0.1.16), while the subsequent analysis was performed using R.

Population structure of the perennial ryegrass association panel was investigated using principal component analysis (PCA). PCA was conducted using “prcomp” function implemented in *stats* package for R. For PCA, genotypic data matrix was used with missing data replaced using mean imputation.

Marker-trait associations were tested using the mean phenotypic values. A GWAS was performed for each trait separately using a single marker regression (SMR, Lenth, 2016) and three complementing methods: (1) FarmCPU (Fixed and random model Circulating Probability Unification, Liu et al., 2016) implemented in GAPIT software package^2^, (2) multi-locus mixed model (MLMM, Segura et al., 2012), and (3) Bayesian-information and Linkage-disequilibrium Iteratively Nested Keyway (BLINK, Zhang et al., 2018) for R. The first three principal components (PC) were provided as covariate variables. For the SMR analysis, each SNP marker was tested with the phenotype as the response variable and the genotype scores as predictor variable. SNPs were retained when more than 100 data points for both the phenotype and the genotype were available. Alleles were removed if their MAF was less than 0.05 If a SNP met these criteria and was still polymorphic, a regression model using the “lm” function in *R* was fitted and the significance (*P*-value) and the variance explained (*R*^2^) was extracted.

To correct for multiple testing, the *P*-values were adjusted using both FDR (Benjamini and Hochberg, 1995) and Bonferroni corrections. An annotation file from the Perennial Ryegrass Genome Sequencing Project (available online at: http://185.45.23.197:5080/ryegrassdata/GENE_ANNOTATIONS/Lp_Annotation_V1.1.mp.gff3) was used to retrieve predicted gene positions and sequence information.

To identify the putative location of markers on chromosome level, scaffolds harboring significant marker-trait associations were anchored to the pseudomolecules of *Hordeum vulgare* genome (Mascher et al., 2017) using LAST^3^.

## Results

### Phenotyping of Leaf Growth Under Water Deficit Conditions

**FIGURE 1:**
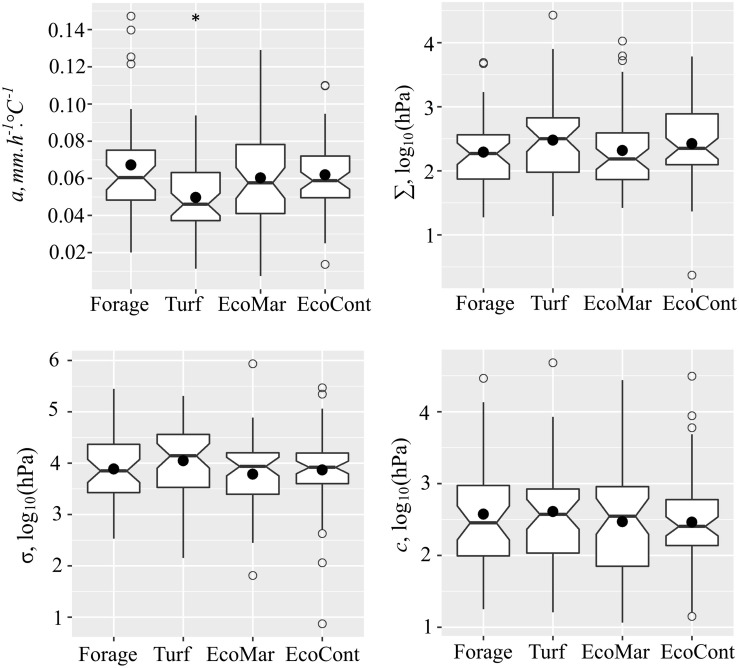
Variation of thermal leaf growth (*a*), leaf growth reduction point (Σ), leaf growth arrest point (σ), and water deprivation tolerance (c) between the forage cultivar (*n* = 43), turf cultivar (*n* = 46), maritime origin ecotype (EcoMar, *n* = 50) and continental origin ecotype (EcoCont, *n* = 58) groups in the perennial ryegrass panel. *indicates significant difference at *P* < 0.05 (Student’s *t*-test).

Substantial variation for leaf growth under water deficit was detected within the perennial ryegrass panel (Figure 1). In the whole association panel, the values for leaf thermal growth rate (*a*) ranged from 0.001 to 0.147 mm.h^–1^.°C^–1^ with a mean *a* value of 0.06 mm.h^–1^.°C^–1^. Growth reduction point (Σ) varied between 1.368–4.432 log_10_ (hPa) with a mean Σ value of 2.38 log_10_ (hPa). The growth arrest point (σ) was lower and varied between 1.87–5.94 log_10_ (hPa) with a mean σ value of 3.89 log_10_ (hPa). The comparison between ecotype and cultivar groups in the association panel revealed no significant differences (*P* > 0.05) for all assessed traits, but significant differences were found when comparing the subgroups. Under optimal conditions, the averaged thermal growth rate of forage type cultivars was *a* = 0.07 mm.h^–1^.°C^–1^, while the turf type cultivars grow significantly (*P* < 0.01) slower with *a* = 0.05 mm.h^–1^.°C^–1^. A significant difference was also observed between the turf subgroup and both ecotype groups originating from maritime and continental climate zones (*P* < 0.05). Overall, the heritability values for the measured traits were high: leaf thermal growth rate (*a*) h^2^ = 0.67, growth reduction point (Σ) h^2^ = 0.72, and growth arrest point (σ) h^2^ = 0.71.

**FIGURE 2:**
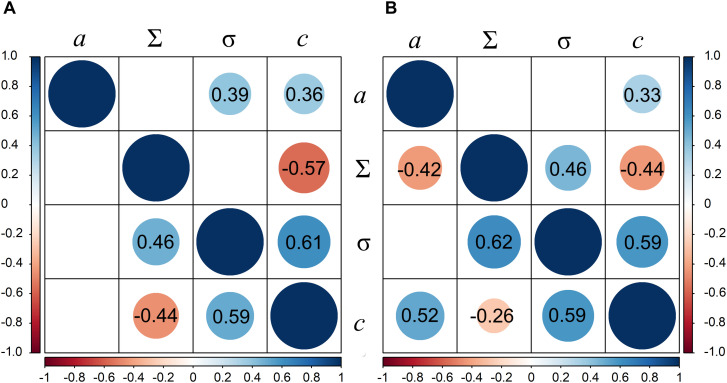
Correlation between thermal leaf growth (*a*), the leaf growth reduction point (Σ), the leaf growth arrest point (σ), and water deprivation tolerance (*c*) traits in perennial ryegrass panel; In **(A)** upper triangle represents forage cultivar group and lower triangle represents turf. **(B)** Upper triangle represents group of ecotypes of maritime origin and lower triangle represents group of ecotypes of continental origin.

Variable correlations between the thermal growth, growth reduction point (Σ), and the growth arrest point (σ) were obtained in the subgroups (Figure 2). Strong to moderate positive and significant correlation was observed between growth reduction and growth arrest points in both ecotype groups and turf type cultivar group, ranging from *r* = 0.62 to *r* = 0.46, *P* < 0.05. Leaf growth arrest point strongly correlated with tolerance of genotypes in all groups (r ranging from 0.50 to 0.61, *P* < 0.05).

### GBS, Population Structure and LD

Sequencing of the GBS libraries yielded over 573 million reads. The number of reads per sample ranged from 257,518 to 3,667,767 reads with an average reads number of 1,528,212 ± 604,999. After removing variant sites with missing rates ≥0.5 and minor allele frequency <0.05, 21,648 markers were identified and used for further analyses.

A rapid LD decay was observed in the perennial ryegrass association panel using GBS-*Pst*I markers. LD was measured by the *r*^2^ and the average distance between the SNPs with LD ≥ 0.5 was determined to be 712 bp. The distance for SNPs with LD ≥ 0.25 was on average 1,378 bp.

**FIGURE 3:**
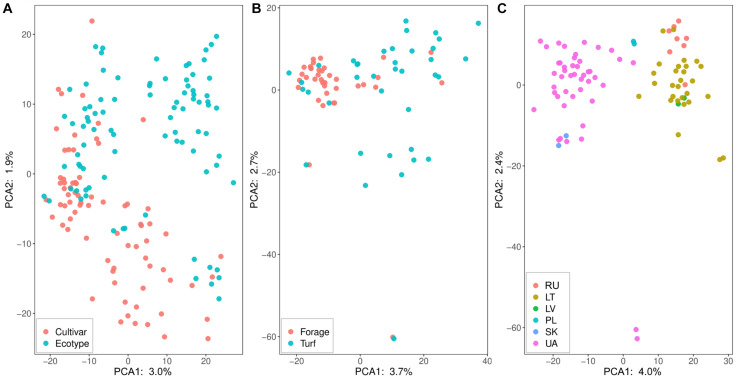
Principal component analysis (PCA) of 197 perennial ryegrass cultivars and ecotypes based on genotyping-by-sequencing data. **(A)** shows the dispersion of cultivars and ecotypes where cultivars are indicated in red and ecotypes in blue dots. The dispersion of cultivars based on the type is shown in **(B)**, where forage type cultivars are represented in red dots and blue dots indicate turf type cultivars. **(C)** demonstrates the distribution of the ecotypes according to their country of origin. Countries are presented in two-letter codes: RU, Kaliningrad region, Russian Federation; LT, Lithuania; LV, Latvia; PL, Poland; SK, Slovakia; and UA, Ukraine.

Genetic structure in perennial ryegrass association panel was visualized using PCA (Figure 3). The first and second PC accounted from 3.0 to 4.0% and from 1.9 to 2.4% of the observed genetic variance, respectively. The first PC (Figure 3A) did not discriminate between ecotypes and cultivars, but the second PC shown some separation. The subgroups of forage and turf type cultivars were not clearly separated (Figure 3B), while for the ecotype group, a clear separation of the genotypes was observed regarding the country of origin (Figure 3C). Moreover, a high correlation was detected between PC1 and geographical latitude (*r* = 0.87, *P* < 0.01). Genotypes for the neighboring countries of Lithuania, Latvia and Kaliningrad region of Russia formed a distinct cluster, while Ukrainian, Polish and Slovakian ecotypes formed a separate cluster. Interestingly, two Ukrainian ecotypes were found to be distantly related to other genotypes with an obvious separation on PC2 (Figure 3C). PC1, in Figure 3C, clearly separated ecotypes originating from the Baltic Sea region from the ecotype group of Ukrainian and Slovakian which represented continental climate zone.

### GWAS for Biomass Formation

**FIGURE 4:**
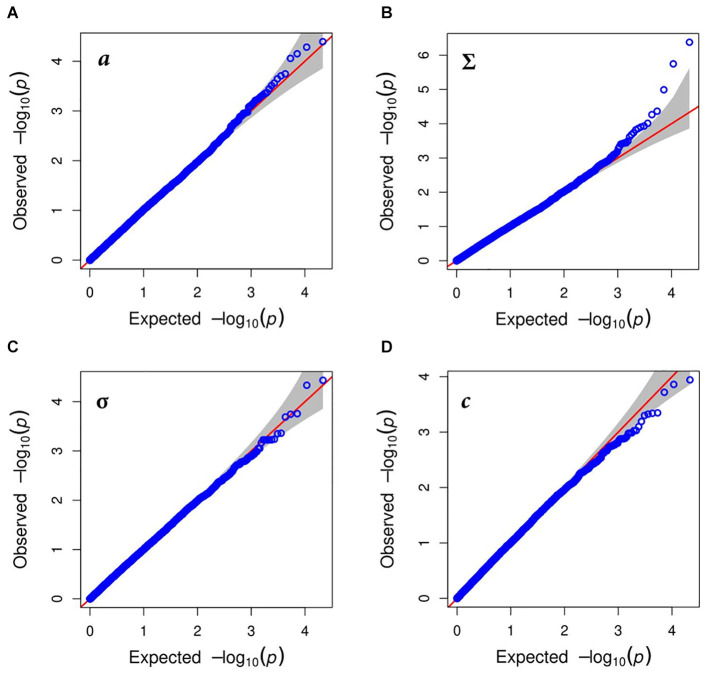
Quantile-quantile (QQ)-plots for biomass formation under water deficit condition traits after correction for population structure: **(A)** shows thermal leaf growth (*a*) of perennial ryegrass under optimal growth conditions; leaf growth reduction point (Σ) presented in **(B)**; in **(C)** the leaf growth arrest point (σ) shown and tolerance to water deprivation (*c*) traits is given in **(D)**.

Marker trait associations were tested individually using SMR and three multi-QTL approaches: FarmCPU, BLINK, and MLMM. No significant marker trait associations were found after correcting for multiple testing, using SMR. This is attributable to the incomplete genotypic data from GBS and rare alleles coupled with strict multiple testing. Although three SNPs were significantly associated with the growth reduction point Σ, after correction for multiple testing, when using FarmCPU, BLINK, and MLMM. These markers are in close proximity or within predicted genes (Table 1 and Figure 4). However, no SNPs were significantly associated with other traits. The significant *MYB41* loci found by FarmCPU had the lowest *P*-value (*P* = 6.35E^–06^) when tested by SMR model. Given that both Bayesian and frequentist procedures, using FarmCPU and SMR statistical approaches, respectively, identified the same loci suggests they are important.

**TABLE 1:** Description of the most significant marker trait associations for the growth decrease trait on genomic scaffolds.

Scaffold	Position	Gene prediction (blastn)	Location	Scaffold position in barley genome	GWAS method	SNP effect	MAF	*P*-value	*P*-values FDR	*P*-values (Bonferroni correction)
scaffold_20866| ref0045961	1878	Transcription factor MYB41 (XM_003573090.4)	Outside gene (708 bp)	Hv_chr6H	FarmCPU	−0.548	0.091	4.19E-07	0.009	0.009
					BLINK	NA	0.091	4.15E-07	0.009	NA
					MLMM	NA	0.091	8.16E-07	0.009	0.018
scaffold_4484| ref0039062	32616	Phytochrome B (XM_020328926.1)	Intron	Hv_chr4H	FarmCPU	0.739	0.054	1.79E-07	0.019	0.039
					BLINK	NA	NA	1.78E-06	0.019	NA
scaffold_21802| ref0017195	728	NA	Intergenic space	NA	MLMM	NA	0.256	4.43E-07	0.009	0.010

*Scaffolds with significant markers were mapped to *Hordeum vulgare* genome assembly.*

The location of the marker-tagged scaffolds at the chromosome level was revealed by BLAST alignment to the barley *H. vulgare* genome (Mascher et al., 2017). Two of the scaffolds which harbored the most significant markers were anchored to chromosomes 4 and 6 in barley. The allele substitution effects for the significant markers associated with growth reduction ranged from −0.548 to 0.739 log10 (hPa). The most significant SNP (*P* = 4.19E^–07^) with an estimated effect size of −0.548 log10 (hPa) was located on a small genomic scaffold 20866| ref0045961 at position 1,878 bp. This scaffold harbored a single gene prediction for *MYB41* transcription factor (TF), while the marker was of 708 bp away from the gene. The second most significant SNP (*P* = 1.79E^–07^), estimated effect of 0.739 log10 (hPa) and was located at 32,616 bp of scaffold 4484| ref0039062 within the intron of predicted gene with a high sequence homology to *Phytochrome B*. The third most significant marker (*P* = 4.436E^–07^) was on scaffold 21802| ref0017195 at position 728 bp. However, there were no gene predictions in close proximity to the marker.

## Discussion

This study revealed the wide range of growth response to water deficit in a diverse panel of perennial ryegrass in a changing environment. The most sensitive genotypes limited the growth already under moist conditions [Σ < 1.50 log_10_ (hPa)], while some of the genotypes maintained leaf growth under lethal conditions [Σ > 4.00 log_10_ (hPa)]. The slower growth of genotypes representing turf cultivars obtained in the study reflects the decades of selective breeding aiming in opposite directions for forage and amenity grasses. The results show that turf types have shorter leaves because they grow slower. Though this may also be attributable to reduced growth duration, which was not evaluated in this study. The shorter, narrower leaves and lower plant height of turf genotypes was already confirmed in previous studies on plant architecture by Statkevičiūtė et al. (2015, 2018). The forage varieties in contrast have been selected for high biomass, for which leaf growth is an important factor (Wilkins, 1991; Barre et al., 2006).

Natural ecotypes are considered a source of stress resistance. Furthermore, they can possess high yielding potential, comparable to that of registered cultivars (Bachmann-Pfabe et al., 2018). The ecotypes in our study demonstrated similar growth patterns to the forage cultivar group. The significant correlation between the growth reduction (Σ) and the growth arrest (σ) points was estimated in all genotype groups except the forage cultivars, but it is worth noting that this correlation was strongest in the continental climate ecotype group. Extensive studies of natural perennial ryegrass diversity in Europe demonstrated that modern cultivars are mostly related to north-western Europe ecotypes (Blanco-Pastor et al., 2019), leaving the vast majority of the natural genetic variation unexploited. This was confirmed in our study as most ecotypes, especially the ones originating from Ukraine, genetically separated from the cultivars with very little overlap. Furthermore, the latitudinal position correlated highly with the first principle component of genetic structure suggesting latitude was a prominent force shaping the diversity of wild-growing perennial ryegrass populations. From a breeder’s perspective, combining an ecotype that responds late to water stress with elite germplasm that has high thermal growth rate would result in a high yielding drought tolerant cultivars.

Three significant marker trait associations were found with growth reduction under water deficit, with all of them passing Bonferroni correction. For two of these markers are in close proximity to predicted genes with sequence homology to *PhytochromeB* (*PhyB*) and *MYB41*. Both genes have well established functions within plant kingdom.

The phytochrome family is a part of the plant circadian clock, they initiate and mediate a complex photomorphogenesis signal cascade when activated by the red light, with the *PhyB* being the main photoreceptor (Nagy and Schäfer, 2002; Srivastava et al., 2019). The role of *PhyB* in plant growth and development has been known for decades when abnormal elongation of various tissues, from stems to root hair, has been demonstrated in *Arabidopsis thaliana phyB* mutant (Reed et al., 1993). Transgenic plants overexpressing *PhyB* had increased transpiration rate caused by higher stomata density and stomatal index, leading to enhanced photosynthesis but reduced water-use efficiency (Boccalandro et al., 2009). The development of rice *phyB* mutants did not differ from WT plants under well-watered conditions; but exhibited higher tolerance when drought stress was applied. This was mainly due to reduced transpiration and stomatal density compared to WT plants (Liu et al., 2012). *PhyB* was also shown to enhance ABA sensitivity under water shortage in *A. thaliana* (González et al., 2012). Moreover, the role of *PhyB* was shown in other abiotic stresses, like heat and cold (He Y. et al., 2016; Song et al., 2017). Plant hormone biosynthesis and signaling pathway genes, as well as abiotic stress-related genes were found to be regulated by *PhyB* in wheat (Pearce et al., 2016).

The MYB proteins comprise one of the largest TF transcription factor families in plants and are known to be involved in multiple functions, including plant development, growth, metabolism, cell fate, and abiotic stress response (Dubos et al., 2010; Baldoni et al., 2015; Li et al., 2015). The R2R3-MYB family, which is the biggest MYB transcription factor family in plants, has been studied in various plant species, especially *Arabidopsis* (Dubos et al., 2010; Du et al., 2012a, b; Katiyar et al., 2012; He et al., 2019). However, not much is known about the MYB TFs in ryegrasses. A number of MYB TFs were demonstrated to coordinate drought and salt stress responses in cotton, sheepgrass and rice with various effects; some were down-regulated during stress, some up-regulated, or differentially expressed in different plant organs (Chen et al., 2015; Zhu et al., 2015; He Q. et al., 2016; Zhao et al., 2019). In *Arabidopsis*, the expression of *MYB41* was only detectable under drought, salt and ABA treatment. Transgenic plants over-expressing this gene were more sensitive to desiccation and had smaller cells among other phenotypic effects. Furthermore, the expression of genes regulating lipid metabolism, cell wall modifications and cell expansion was altered (Cominelli et al., 2008). The increased expression of *MYB41* in osmotic stress response was confirmed in a study by Lippold et al. (2009), although the effect of *MYB41* over-expression on plant growth were less drastic. Involvement of *MYB41* in cell-wall associated lipid metabolism as well as activation of *MYB41* promoter in the endodermis under abiotic stress was observed in both *Arabidopsis* and *Nicotiana benthamiana* (Kosma et al., 2014). These results suggest that *MYB41* might confer osmotic stress resistance by regulating cell wall and leaf cuticle deposition, yet the exact mode of action of this particular TF is not defined due to the complexity of the gene regulation network it is involved in. A number of other MYB TFs modulate plant stress resistance in a similar manner to *PhyB* by regulating size, density, and opening rates of stomata (Baldoni et al., 2015; Chen et al., 2015; Butt et al., 2017).

Taken together, this study combines precision phenotyping of leaf growth in response to water stress, with genome wide association. The *PhyB* and *MYB41* genes, identified in this study, are known to modulate abiotic stress response in many plant species. Given the sub-gene LD level in this population, suggests they are the causal genes underlying the detected QTL. To our knowledge this is the first time these genes have been associated with perennial ryegrass leaf growth under drought stress. The study also has practical applications for plant breeding; depicting how different, natural, alleles might be used to improve drought tolerance and what effect they have. In addition, the genes found highlight the potential of the phenotyping method. Namely, that using leaf growth as a proxy for stress response is a tool that can be used to dissect a complex trait into fundamental components. Thus, improving our understanding of plant behavior under water stress and subsequently, yielding potential in the regions experiencing frequent albeit mild summer droughts. Cultivars, purposely selected for a high tolerance, in terms of growth reduction and arrest by our phenotyping model, might provide a reliable feed source in the changing environment of the future world.

## Data Availability Statement

The data is shared on https://doi.org/10.6084/m9.figshare.12433799.v3 and https://doi.org/10.6084/m9.figshare.12433715.v3.

## Author Contributions

KJ, SY, and BS conceived the study. SY and KJ conducted the experiments. KJ, AA, and SY performed data analysis. VK and GS assisted in data analysis and interpretation. KJ, GS, and AA drafted the manuscript, which was improved by SY, VK, and BS. All authors read and approved the final manuscript.

## Conflict of Interest

The authors declare that the research was conducted in the absence of any commercial or financial relationships that could be construed as a potential conflict of interest.

## Footnotes

1 https://github.com/najoshi/sabre

2 https://github.com/jiabowang/GAPIT3

3 http://last.cbrc.jp/
