# Supplementary material for: Genome-Wide Association Study to Identify Candidate Loci for Biomass Formation Under Water Deficit in Perennial Ryegrass
Source: Front Plant Sci. 2020 Dec 8;11:570204. doi: 10.3389/fpls.2020.570204 (PMC7841438; doi:10.3389/fpls.2020.570204)
Supplement: Supplementary file 1 [file Table_1.DOCX]

Supplementary Material

# Supplementary Tables

**Supplementary Table 1.** List of the cultivars represented in the association panel used to study leaf growth under water deficit in perennial ryegrass (*Lolium perenne* L.).

| **Genotype number** | **Country of origin** | **Cultivar name** | **Type** |
| --- | --- | --- | --- |
| 1 | Belgium | Vigor | forage |
| 2 | Switzerland | Arvella | forage |
| 3 | Germany | Kerem | forage |
| 4 | Germany | Loretta | turf |
| 5 | Germany | Lisuna | turf |
| 6 | Germany | Lorina | turf |
| 7 | Germany | Marietta | turf |
| 8 | Germany | Langa | turf |
| 9 | Germany | Excel | turf |
| 10 | Germany | Fancy | turf |
| 11 | Germany | Juwel | turf |
| 12 | Germany | Livonne | turf |
| 13 | Germany | Trimmer | turf |
| 14 | Denmark | Pimpernel | forage |
| 15 | Denmark | Taya | turf |
| 16 | Denmark | Eden | turf |
| 17 | Denmark | Sedona | turf |
| 18 | Denmark | Belida | forage |
| 19 | Denmark | Danilo | forage |
| 20 | Denmark | Sydney | forage |
| 21 | Denmark | Sakini | turf |
| 22 | Denmark | Dacapo | turf |
| 23 | Finland | Riikka | forage |
| 24 | France | Barcredo | turf |
| 25 | France | Dragon | turf |
| 26 | Hungary | Karcagi | forage |
| 27 | Japan | Kryosato | forage |
| 28 | Lithuania | Veja DS | forage |
| 29 | Latvia | Priekulskij 59 | forage |
| 30 | Netherlands | Toronto | forage |
| 31 | Netherlands | Wendy | forage |
| 32 | Netherlands | Morgana | forage |
| 33 | Netherlands | Amigo | forage |
| 34 | Netherlands | Bravo | forage |
| 35 | Netherlands | Duramo | forage |
| 36 | Netherlands | Trani | forage |
| 37 | Netherlands | Gladio | forage |
| 38 | Netherlands | Bologna | turf |
| 39 | Netherlands | Regal | turf |
| 40 | Netherlands | Aurelius | turf |
| 41 | Netherlands | Barluxe | turf |
| 42 | Netherlands | Barmona | turf |
| 43 | Netherlands | Barrage | turf |
| 44 | Netherlands | Boston | turf |
| 45 | Netherlands | Cherokee | turf |
| 46 | Netherlands | Dali | turf |
| 47 | Netherlands | Darius | turf |
| 48 | Netherlands | Entar | turf |
| 49 | Netherlands | Jubilar | turf |
| 50 | Netherlands | Master | turf |
| 51 | Netherlands | Numan | turf |
| 52 | Netherlands | Ovation | turf |
| 53 | Netherlands | Perfect | turf |
| 54 | Netherlands | Rival | turf |
| 55 | Netherlands | Sensation | turf |
| 56 | Netherlands | Surprise | turf |
| 57 | Netherlands | Troubadour | turf |
| 58 | Netherlands | Heraut | forage |
| 59 | Netherlands | Kerdion | forage |
| 60 | Netherlands | Magella | forage |
| 61 | Netherlands | Magyar | forage |
| 62 | Netherlands | Mongita | forage |
| 63 | Netherlands | Morenne | forage |
| 64 | Netherlands | Moronda | forage |
| 65 | Netherlands | Peramo | forage |
| 66 | Netherlands | Prefence | forage |
| 67 | Netherlands | Premium | forage |
| 68 | Netherlands | Recolta | forage |
| 69 | Netherlands | Salem | forage |
| 70 | Netherlands | Summit | forage |
| 71 | Netherlands | Veritas | forage |
| 72 | Netherlands | Bardonna | forage |
| 73 | Netherlands | Barclay | turf |
| 74 | Netherlands | Blazer | turf |
| 75 | New Zealand | Grasslands Nui | forage |
| 76 | Poland | Anna | forage |
| 77 | Poland | Naki | forage |
| 78 | Poland | Gazon | turf |
| 79 | Poland | Niga | turf |
| 80 | Poland | Arka | forage |
| 81 | Russia | Leningradskij 809 | forage |
| 82 | Russia | Siverskij 809 | forage |
| 83 | Sweden | Svea | forage |
| 84 | Sweden | Ronja | turf |
| 85 | USA | Barloft | turf |
| 86 | USA | Wizard | turf |
| 87 | USA | Linn | forage |
| 88 | USA | Manhattan | turf |
| 89 | USA | Pennfine | turf |
| 90 | USA | Repell | forage |

**Supplementary Table 2.** A detailed description of the ecotypes represented in the association panel used to study leaf growth under water deficit in perennial ryegrass (*Lolium perenne* L.). NA; data not available.

| **Genotype number** | **Country of origin** | **Name** | **Subgroup** | **Latitude** | **Longitude** | **Elevation (m)** |
| --- | --- | --- | --- | --- | --- | --- |
| 1 | Lithuania | ecotype | maritime | NA | NA | NA |
| 2 | Lithuania | ecotype | maritime | 55.27 | 21.47 | 1,00 |
| 3 | Lithuania | ecotype | maritime | NA | NA | NA |
| 4 | Lithuania | ecotype | maritime | 55.22 | 21.53 | 6,00 |
| 5 | Lithuania | ecotype | maritime | 55.40 | 24.03 | 31,00 |
| 6 | Lithuania | ecotype | maritime | 55.53 | 24.10 | 38,00 |
| 7 | Lithuania | ecotype | maritime | 55.35 | 24.02 | 44,00 |
| 8 | Lithuania | ecotype | maritime | 55.43 | 23.65 | 84,00 |
| 9 | Lithuania | ecotype | maritime | NA | NA | NA |
| 10 | Lithuania | ecotype | maritime | 54.58 | 24.55 | 166,00 |
| 11 | Lithuania | ecotype | maritime | NA | NA | NA |
| 12 | Lithuania | ecotype | maritime | 55.58 | 24.97 | 88,00 |
| 13 | Lithuania | ecotype | maritime | 55.68 | 24.98 | 102,00 |
| 14 | Lithuania | ecotype | maritime | 55.50 | 23.52 | 127,00 |
| 15 | Lithuania | ecotype | maritime | 55.50 | 23.48 | 120,00 |
| 16 | Lithuania | ecotype | maritime | 55.80 | 23.87 | 80,00 |
| 17 | Lithuania | ecotype | maritime | NA | NA | NA |
| 18 | Lithuania | ecotype | maritime | 56.24 | 24.78 | 48,00 |
| 19 | Lithuania | ecotype | maritime | 54.16 | 24.19 | 116,00 |
| 20 | Lithuania | ecotype | maritime | 55.08 | 25.67 | 145,00 |
| 21 | Lithuania | ecotype | maritime | 55.09 | 26.07 | 180,00 |
| 22 | Lithuania | ecotype | maritime | 55.16 | 26.22 | 208,00 |
| 23 | Lithuania | ecotype | maritime | NA | NA | NA |
| 24 | Lithuania | ecotype | maritime | 56.30 | 23.39 | 67,00 |
| 25 | Lithuania | ecotype | maritime | 56.36 | 23.27 | 80,00 |
| 26 | Lithuania | ecotype | maritime | NA | NA | NA |
| 27 | Lithuania | ecotype | maritime | 56.30 | 23.13 | 80,00 |
| 28 | Lithuania | ecotype | maritime | 56.22 | 22.56 | 58,00 |
| 29 | Lithuania | ecotype | maritime | 56.17 | 22.74 | 75,00 |
| 30 | Lithuania | ecotype | maritime | 56.03 | 22.93 | 99,00 |
| 31 | Lithuania | ecotype | maritime | NA | NA | NA |
| 32 | Lithuania | ecotype | maritime | 54.45 | 22.85 | 221,00 |
| 33 | Lithuania | ecotype | maritime | 54.42 | 22.93 | 231,00 |
| 34 | Lithuania | ecotype | maritime | 54.56 | 22.77 | 120,00 |
| 35 | Lithuania | ecotype | maritime | 54.48 | 23.38 | 76,00 |
| 36 | Latvia | ecotype | maritime | 56.50 | 23.36 | 23,00 |
| 37 | Latvia | ecotype | maritime | 57.07 | 22.54 | 38,00 |
| 38 | Poland | ecotype | maritime | 49.38 | 21.70 | 433,00 |
| 39 | Poland | ecotype | maritime | 51.69 | 20.97 | 133,00 |
| 40 | Russian Federation | ecotype | maritime | 54.74 | 20.05 | 5,00 |
| 41 | Russian Federation | ecotype | maritime | 54.52 | 20.01 | 16,00 |
| 42 | Russian Federation | ecotype | maritime | 54.50 | 20.11 | 69,00 |
| 43 | Russian Federation | ecotype | maritime | 54.66 | 20.37 | NA |
| 44 | Russian Federation | ecotype | maritime | 54.58 | 20.47 | 18,00 |
| 45 | Russian Federation | ecotype | maritime | 54.56 | 20.47 | 12,00 |
| 46 | Russian Federation | ecotype | maritime | 54.49 | 20.45 | 47,00 |
| 47 | Russian Federation | ecotype | maritime | 54.48 | 20.93 | 25,00 |
| 48 | Russian Federation | ecotype | maritime | NA | NA | NA |
| 49 | Russian Federation | ecotype | maritime | NA | NA | NA |
| 50 | Slovakia | ecotype | continental | 48.72 | 21.81 | 178,00 |
| 51 | Slovakia | ecotype | continental | 49.14 | 21.54 | 249,00 |
| 52 | Ukraine | ecotype | continental | 48.28 | 24.37 | 697,00 |
| 53 | Ukraine | ecotype | continental | 48.16 | 24.29 | 616,00 |
| 54 | Ukraine | ecotype | continental | NA | NA | NA |
| 55 | Ukraine | ecotype | continental | NA | NA | NA |
| 56 | Ukraine | ecotype | continental | 48.21 | 23.15 | 289,00 |
| 57 | Ukraine | ecotype | continental | 50.61 | 24.96 | 212,00 |
| 58 | Ukraine | ecotype | continental | 49.98 | 24.71 | 224,00 |
| 59 | Ukraine | ecotype | continental | 49.98 | 24.93 | 221,00 |
| 60 | Ukraine | ecotype | continental | NA | NA | NA |
| 61 | Ukraine | ecotype | continental | NA | NA | NA |
| 62 | Ukraine | ecotype | continental | 50.61 | 26.48 | 240,00 |
| 63 | Ukraine | ecotype | continental | NA | NA | NA |
| 64 | Ukraine | ecotype | continental | 50.57 | 27.21 | 217,00 |
| 65 | Ukraine | ecotype | continental | 50.58 | 27.72 | 208,00 |
| 66 | Ukraine | ecotype | continental | 50.58 | 27.72 | 208,00 |
| 67 | Ukraine | ecotype | continental | 50.46 | 28.09 | 216,00 |
| 68 | Ukraine | ecotype | continental | 50.35 | 28.34 | 223,00 |
| 69 | Ukraine | ecotype | continental | 50.53 | 28.68 | 200,00 |
| 70 | Ukraine | ecotype | continental | 50.67 | 28.63 | 200,00 |
| 71 | Ukraine | ecotype | continental | 50.73 | 28.63 | 181,00 |
| 72 | Ukraine | ecotype | continental | 50.99 | 28.50 | 194,00 |
| 73 | Ukraine | ecotype | continental | 50.80 | 27.96 | 204,00 |
| 74 | Ukraine | ecotype | continental | 50.58 | 26.24 | 186,00 |
| 75 | Ukraine | ecotype | continental | 50.48 | 25.85 | 245,00 |
| 76 | Ukraine | ecotype | continental | NA | NA | NA |
| 77 | Ukraine | ecotype | continental | 51.04 | 25.12 | 174,00 |
| 78 | Ukraine | ecotype | continental | 49.49 | 23.36 | 302,00 |
| 79 | Ukraine | ecotype | continental | 49.44 | 23.41 | 295,00 |
| 80 | Ukraine | ecotype | continental | 49.44 | 23.41 | 295,00 |
| 81 | Ukraine | ecotype | continental | 49.39 | 23.31 | 366,00 |
| 82 | Ukraine | ecotype | continental | 49.27 | 23.30 | 569,00 |
| 83 | Ukraine | ecotype | continental | 49.17 | 23.39 | 669,00 |
| 84 | Ukraine | ecotype | continental | 49.23 | 23.29 | 632,00 |
| 85 | Ukraine | ecotype | continental | 49.22 | 23.29 | 534,00 |
| 86 | Ukraine | ecotype | continental | 49.74 | 23.66 | 256,00 |
| 87 | Ukraine | ecotype | continental | 49.94 | 23.97 | 373,00 |
| 88 | Ukraine | ecotype | continental | NA | NA | NA |
| 89 | Ukraine | ecotype | continental | 50.11 | 23.89 | 244,00 |
| 90 | Ukraine | ecotype | continental | 50.55 | 24.38 | 239,00 |
| 91 | Ukraine | ecotype | continental | NA | NA | NA |
| 92 | Ukraine | ecotype | continental | NA | NA | NA |
| 93 | Ukraine | ecotype | continental | 51.35 | 24.22 | 185,00 |
| 94 | Ukraine | ecotype | continental | 51.55 | 24.09 | 161,00 |
| 95 | Ukraine | ecotype | continental | 51.40 | 24.10 | 162,00 |
| 96 | Ukraine | ecotype | continental | 51.89 | 26.61 | 161,00 |
| 97 | Ukraine | ecotype | continental | 51.37 | 26.77 | 166,00 |
| 98 | Ukraine | ecotype | continental | 51.22 | 26.49 | 147,00 |
| 99 | Ukraine | ecotype | continental | 50.12 | 27.24 | 278,00 |
| 100 | Ukraine | ecotype | continental | 50.03 | 27.33 | 255,00 |
| 101 | Ukraine | ecotype | continental | 49.78 | 27.35 | 279,00 |
| 102 | Ukraine | ecotype | continental | 49.55 | 26.35 | 261,00 |
| 103 | Ukraine | ecotype | continental | NA | NA | NA |
| 104 | Ukraine | ecotype | continental | 49.64 | 25.37 | 358,00 |
| 105 | Ukraine | ecotype | continental | NA | NA | NA |
| 106 | Ukraine | ecotype | continental | 49.70 | 27.35 | 304,00 |
| 107 | Ukraine | ecotype | continental | 49.74 | 26.18 | 311,00 |
